# Recent Progress in Preparation, Improvement and Applications of Green Polymer Composites

**DOI:** 10.1002/bip.70059

**Published:** 2025-11-08

**Authors:** Kobe Samuel Mojapelo, Williams Kehinde Kupolati, Everardt Andre Burger, Julius Musyoka Ndambuki, Jacques Snyman, Emmanuel Rotimi Sadiku

**Affiliations:** ^1^ Department of Civil Engineering Tshwane University of Technology Pretoria South Africa; ^2^ Institute for NanoEngineering Research (INER) and Department of Chemical Metallurgy and Materials Engineering Tshwane University of Technology Pretoria South Africa

**Keywords:** biodegradable polymers, green polymer composites, mechanical properties, nanoparticle

## Abstract

Green polymer composites (GPCs) are increasingly recognised as sustainable alternatives to traditional petroleum‐based materials, effectively addressing critical environmental challenges, such as plastic pollution and carbon emissions. These composites include biodegradable polymers, natural fibres, and nanomaterials, which enhance their mechanical properties, durability, and eco‐friendly disposal options. However, their widespread industrial adoption faces challenges related to cost, scalability, fibre‐matrix compatibility, and regulatory compliance. This study provides a comprehensive review of recent advancements in GPCs, focusing on the preparation methods, reinforcement strategies, and significant performance improvements. Techniques such as melt blending, compression moulding, and additive manufacturing have notably enhanced interfacial bonding and thermal stability. Comparative analyses indicate that GPCs can achieve up to a 30% increase in tensile strength and a 40% reduction in carbon footprint compared with conventional composites. Despite these advantages, ongoing concerns regarding manufacturing costs, processing limitations, and recyclability highlight the necessity for further optimisation. GPCs have diverse applications in various industries, including the automotive sector, biomedicine and scaffolds, packaging and coatings, and constructing reinforced polymer composites for structural applications. Practical implementation must overcome cost barriers and ensure compliance with the global sustainability regulations. Future research should prioritise enhancing the economic viability of GPCs and conducting life cycle assessments (LCA).

## Graphical abstract

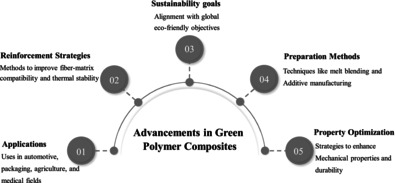

## 1 Introduction

Petroleum‐based polymers have raised serious environmental concerns such as plastic pollution, resource depletion, and greenhouse gas emissions from fossil fuel extraction and processing, which have led to questions about their continuous use [1, 2]. To tackle this challenge, researchers are concentrating on green polymer composites (GPCs), matrices composed of biodegradable polymers, natural fibres, and nanomaterials, as more sustainable alternatives to synthetic materials. Such composites contribute to a greener world as they increase environmental performance, while their mechanical properties remain competitive [3]. GPCs often utilise biopolymers such as polylactic acid (PLA) and polyhydroxyalkanoates (PHA) in combination with jute and flax to significantly enhance material strength and biodegradability [4]. Although design optimisations and new formula developments hold promise for the future of GPCs, there are still key challenges to overcome in terms of cost efficiency, scalability, fibre‐matrix interactions, and regulation compliance before these materials are widely adopted [5]. Future studies are needed to overcome these limitations using novel strategies such as the use of functionalised nanomaterials and hybrid reinforcement. AI‐based frameworks can also play a prominent role in optimising processing conditions for improved material performance and sustainability [6]. The journey towards commercialisation also relies on investments in cost‐efficient production techniques, optimised supply chains, and the development of more integrated testing frameworks for regulatory compliance and sustainability [7]. Manufacturing techniques underlie the progression of GPC technology from melt blending to AM. The use of novel hybrid materials and the introduction of nanofillers play a significant role in attaining both optimal properties and good compatibility [8]. Furthermore, the automotive, construction, biomedical, and packaging sectors benefit from diverse applications of GPCs, making them an alternative to traditional composite materials [9]. Emphasis has been placed on long‐term durability, LCA, and standardisation to support the broader commercialisation of sustainable polymer technologies [10].

**FIGURE 1:**
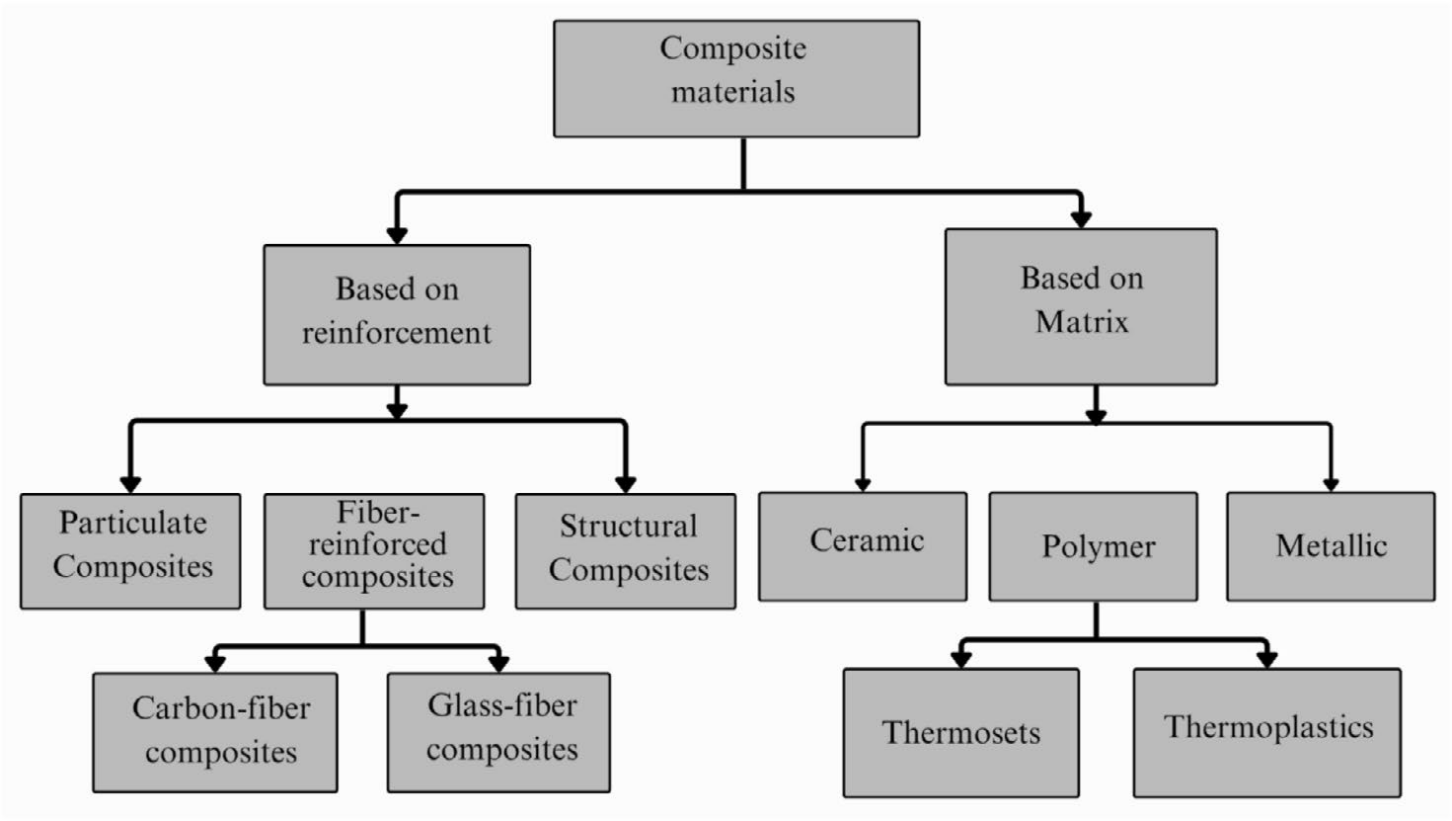
Classification of composites based on reinforcement and matrix types [11].

The classification system for composite materials according to reinforcement and matrix type is shown in Figure 1. In terms of reinforcement, composites are categorized into particulate, fibre‐reinforced design, and structural composites, whereas fibre‐reinforced composites are divided into natural fibre and synthetic fibre categories. Composites can be classified as ceramic, polymer, and metallic matrices, whereas polymer matrices are split into thermosets and thermoplastics. The framework reflects the structural diversity of the composite, allowing tailored properties for specific applications. This review critically evaluates the recent progress in the development, processing, and application of GPCs, focusing on performance optimization, environmental impact, and industrial feasibility.

A brief comparison of GPCs and conventional materials is presented in Table 1. GPCs can deliver a lower impact on the environment, lower carbon dioxide (CO_2_) emissions, higher recyclability, and better biodegradability owing to the presence of natural fibres and renewable matrices. They also minimise waste by using agricultural waste and recycled materials, which reduces the consumption of primary resources. GPCs may offer durability comparable to that of lithium‐ion batteries, but they are more energy efficient. Alternatively, traditional materials depend on non‐renewable resources, cannot be reused, produce higher emissions, and incur higher disposal costs.

**TABLE 1:** Comparative analysis of green polymer composites and conventional materials.

Aspect	Green polymer composites	Conventional materials	Sources
CO_2_ emissions	Reduced CO_2_ emissions due to natural fibre content and renewable matrices	Higher CO_2_ emissions from synthetic fibres and petroleum‐based polymers	[2]
Recyclability	High recyclability, particularly with thermoplastics like PLA and PHA	Limited recyclability, often ending in landfills or incineration	[12]
Biodegradability	Enhanced biodegradability with natural fillers such as hemp, jute, and bamboo	Non‐biodegradable synthetic composites	[13]
Resource consumption	Reduced reliance on fossil fuels by incorporating renewable materials such as lignocellulosic fibres	High resource consumption due to dependence on non‐renewable inputs	[14]
Durability	Comparable or improved durability when optimised with natural and hybrid fillers	Generally durable but environmentally taxing	[15]
Waste utilization	It incorporates waste materials such as agricultural by‐products (e.g., rice husk) and recycled polymers (e.g., polyethylene terephthalate (PET) bottles)	Rarely incorporates waste and has high environmental disposal costs	[16]
Energy efficiency	Lower energy requirements during production for some types	Higher energy demands during material synthesis	[17]

## 2 Processing Techniques and Their Influence on Composite Microstructure and Performance

Biodegradable polymers combined with natural fibres and bio‐based fillers have been used to prepare green polymeric composites with improved mechanical, thermal, and functional properties [18]. Natural fillers, such as nanocellulose, flax, and kenaf, enhance tensile strength and thermal stability  [19]. However, because hydrophilic fibres and hydrophobic polymers do not have a particular affinity, surface treatments and compatibilisers are required [20]. Processing techniques such as melt compounding, solvent casting, and electrospinning tailor the composite architecture and fibre dispersion. Melt mixing allows for homogeneous fibre dispersion in the polymer matrix under high shear, possibly leading to fibre agglomeration and thermal degradation. Solvent casting preserves fibre integrity but can cause phase separation and porosity, affecting the mechanical homogeneity. Electrospinning, which is common in the biomedical field, enables nanoscale fibre alignment to enhance the tensile strength and bonding surface area, but lacks scalability [21].

The microstructure of natural fibre composites is significantly modified through processing techniques, which are characterised by instrumentation methods. Alkaline or silane treatment shows better fibre‐matrix locking and fewer voids, as observed in scanning electron microscope (SEM) results. Improved adhesion is achieved due to increased surface roughness [22, 23]. Fourier Transform Infrared (FTIR) spectra corroborate chemical functionalisation with silane modifications, which promote fibre‐polymer compatibility and the augmentation of mechanical properties [24]. X‐Ray diffraction (XRD) indicates that optimal melt blending and crosslinking could lead to higher crystallinity and structural orientation of the composites, corresponding to better stiffness and phase transformation [25, 26]. Advanced approaches, such as controlling the molecular weight and incorporating nanofillers such as graphene quantum dots and silver nanoparticles, further enhance performance and impart additional functions, including antimicrobial resistance [27]. These microstructural refinements contribute to the enhanced mechanical, thermal, and functional properties required for advanced composite applications.

Surface alterations (physical defibrillation, chemical silane, and alkali) and combination fillers (Graphene Quantum Dots and silver nanoparticles) increase adhesion, mechanical features, and antimicrobial properties. New methods, such as cross‐linking, hybrid reinforcement, and molecular weight control, can be customised for use in fields such as construction and packaging. The updated classification of green polymer composites based on the types of reinforcements, type of matrix material used, and application areas is listed in Table 2. Natural fibres (jute, flax, hemp, etc.), nanoparticles (graphene oxide and nanoclays), hybrids, and agricultural and recycled fibres are among the reinforcements used to enhance strength, durability, and sustainability. Matrices have been selected for their green properties, ranging from biodegradable polymers such as polylactic acid (PLA) and polyhydroxyalkanoate (PHA) to recycled plastics, hybrids, natural resins, and bio‐based thermosets. These composites can be utilised in various industries, such as construction, automotive, packaging, agriculture, medicine, and aerospace, highlighting their versatility and increasing significance in developing sustainable material solutions.

**TABLE 2:** Classification of green polymer composites.

Category	Type	Details	Citations
Reinforcements	Natural fibres	Jute, hemp, flax, sisal, coir, bamboo, kenaf	[28]
	Nanoparticles	Nano‐clay, graphene oxide, carbon nanotubes, cellulose nanocrystals	[29]
	Hybrid materials	Combination of natural fibres and nanoparticles (e.g., flax + graphene oxide, jute + SiO_2_)	[30]
	Agricultural waste	Rice husk, date palm fibres, sugarcane bagasse	[31]
	Recycled fibres	Recycled glass fibres, pet bottle fibres	[32]
Matrices	Biodegradable polymers	Polylactic acid (PLA), polyhydroxyalkanoates (PHA), alginate	[33]
	Recycled plastics	Recycled Polypropylene (PP), Recycled PET, Recycled ABS	[34]
	Hybrid polymers	Blends of biodegradable and recycled polymers (e.g., PLA/PBS blends)	[21]
	Natural resins	Soy‐based resins, epoxidized linseed oil	[35]
	Bio‐based thermosets	Lignin‐based epoxy, chitosan‐based polymers	[36]
Applications	Construction	Partition walls, insulation panels, load‐bearing structures, roofing materials	[37]
	Automotive	Lightweight components for vehicles, dashboards, and electric vehicle parts	[38]
	Packaging	Biodegradable films, eco‐friendly packaging solutions, barrier coatings	[39]
	Agriculture	Soil retention, water‐saving composites, and biodegradable crop protection materials	[40]
	Medical	Biodegradable implants, drug delivery systems, wound healing materials, tissue engineering scaffolds	[41]
	Aerospace	Lightweight aircraft components, vibration dampers, thermal insulation	[42]

### 2.1 Melt Blending

Melt blending is a crucial method for fabricating homogeneous polymer green composites by mixing thermoplastic polymers with natural fibres and fillers at elevated temperatures using extruders. As the polymer is heated, its viscous nature decreases, which supports mechanical shear forces to promote the dispersion of the fibres and interfacial contact. While higher temperatures enhance mixing, they may cause fibre charring. Lower temperatures can improve the coating uniformity while reducing the bond and layer strengths. Highly controllable processing of fibre‐polymer mixtures is key to twin‐screw extruders, primarily when adhesion promoters and surface treatments are used [43], which promotes sustainable manufacturing practices by using renewable fibres from agricultural waste through an environmentally friendly, solvent‐free, and low‐emission process.

### 2.2 Compression Moulding

**FIGURE 2:**
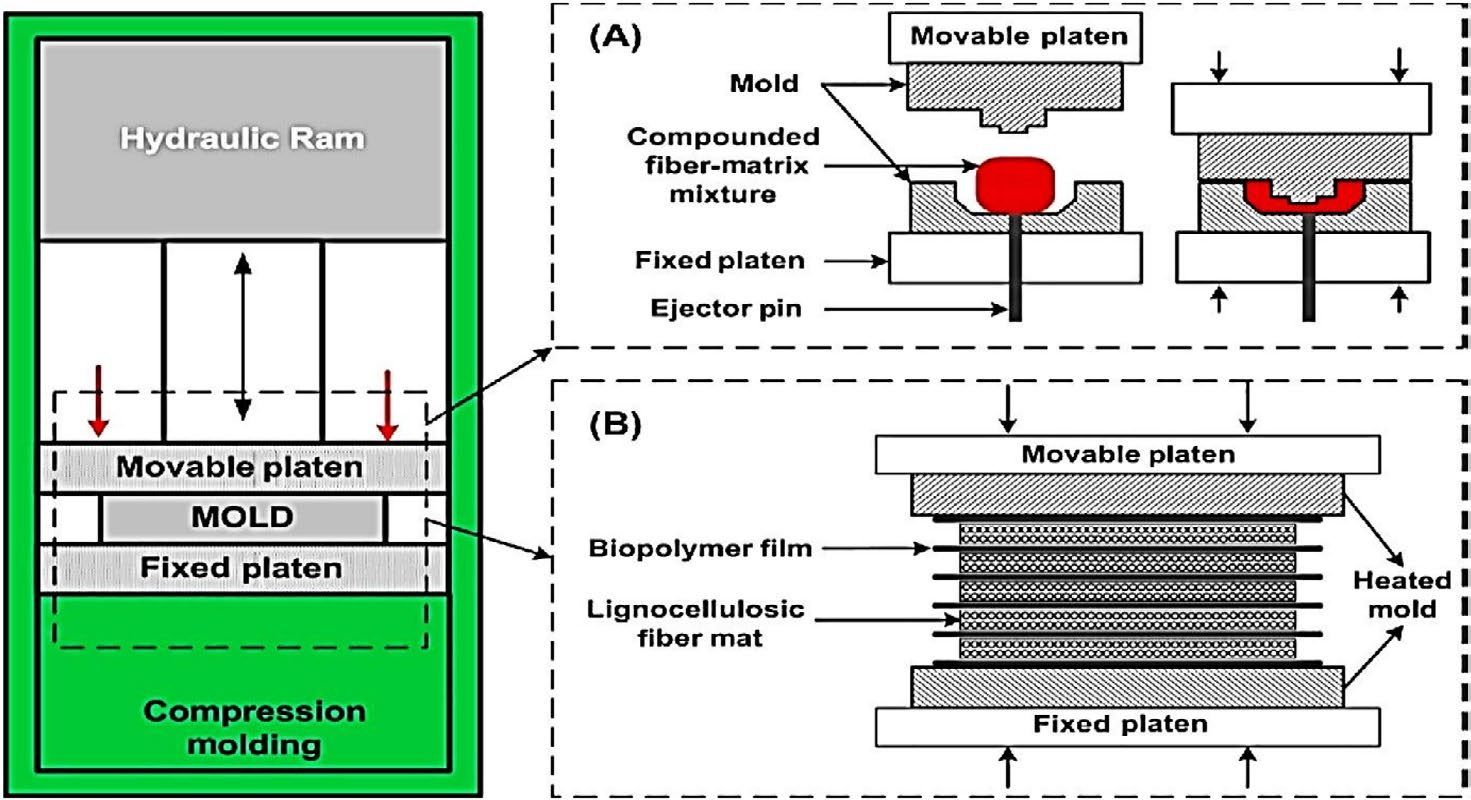
Schematic of the compression moulding process [47].

Various techniques have been employed to manufacture green polymer composites, including compression moulding, which is a commonly used method for combining polymers with natural fillers and advanced materials [44]. This procedure promotes sustainability by utilising biodegradable and recycled materials, minimising waste through recycling, and decreasing reliance on virgin polymers [45]. The performance at relatively low temperatures preserves the structural integrity of the sensitive fillers while enhancing their mechanical, thermal, and electrical properties. Green polymer composites have applications in electronics for electromagnetic interference (EMI) shielding, and lightweight and durable materials are essential in the automotive and consumer product sectors [46]. In compression moulding, heat and pressure are used together for amorphous polymers, as shown in Figure 2. Panel A illustrates a fibre matrix mixture packed into a mould cavity between a movable and fixed platen. The hydraulic ram applies pressure to the polymer, transitioning it from solid to soft and allowing it to flow around the fillers and trap them, whereas the ejector pin facilitates demoulding. Panel B illustrates an alternative setup that utilises biopolymer films and lignocellulosic fibre mats. This heat and pressure create homogeneous bonding and eliminates voids in the composite, resulting in a high‐quality, dense material in a single process. However, achieving specific properties of the composite requires precise control of temperature, pressure, and time.

### 2.3 Injection Moulding

**FIGURE 3:**
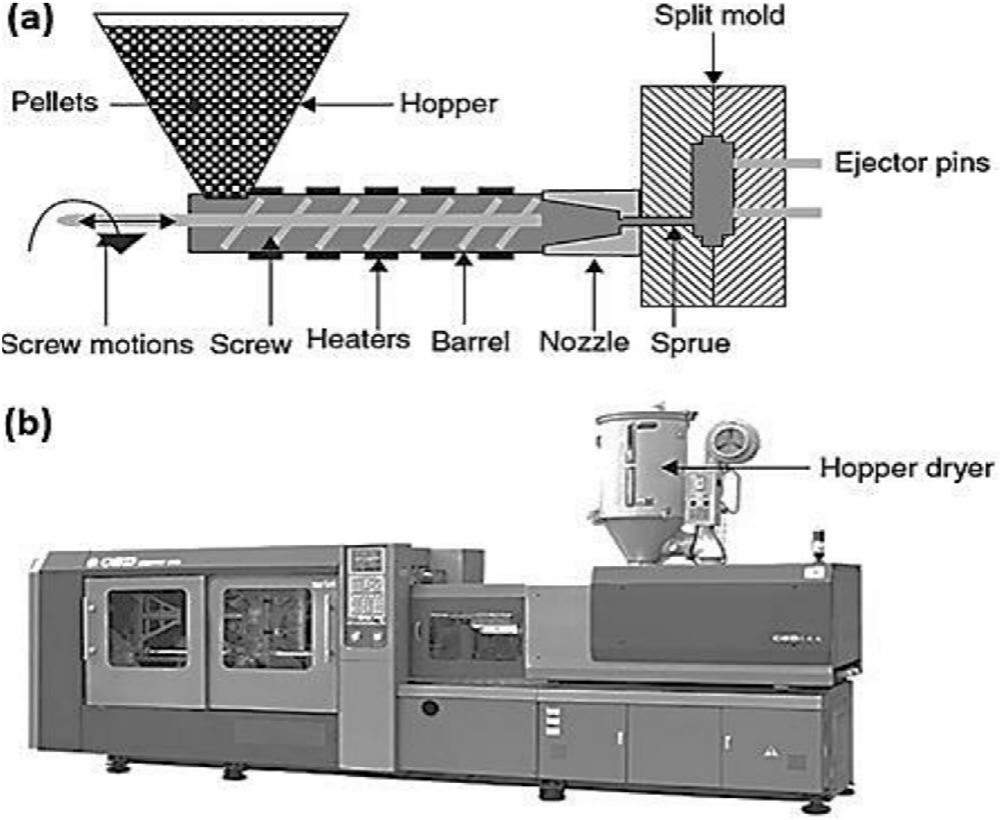
Injection moulding machine setup: (a) Schematic injection moulding setup and (b) injection moulding setup with a hopper [51].

Zhang et al. [48] characterised injection moulding as an effective and widely used method for producing environmentally friendly green polymer composites. This process begins with the creation of a polymer‐filler blend, ensuring an even distribution of the filler throughout the polymer matrix through techniques such as melt blending or dry blending. The mixture then passes through a heated barrel, melting to a flowable state, as shown in Figure 3. A screw mechanism injects molten material into a prefabricated mould cavity at a high pressure, ensuring that the mixture fills intricate geometries and fine details. The combination of increased heat and pressure promotes strong interfacial bonding between the polymer and filler, thereby improving the mechanical properties of the composite. Waalkes et al. [49] emphasised the need for precise control of the processing parameters in injection moulding to optimise the performance of green polymer composites. Key factors such as injection pressure, melt temperature, cooling time, and packing pressure significantly influence the final properties of the moulded composite. Maintaining the optimal injection speed and pressure is critical to ensure complete mould filling, thereby minimising defects, such as voids, weld lines, and distortion. Additionally, advanced techniques, such as water‐assisted injection and gas‐assisted moulding, improve the process by enhancing filler orientation and distribution. These innovations improve both the mechanical and thermal properties and reduce the material and energy consumption, thereby aligning with sustainability goals. Katogi [50] highlights recent advances in moulding technology and material formulations that have expanded the possibilities of injection moulding for green polymer composites.

### 2.4 Hand Lay‐Up Method

The hand‐lay‐on method is a basic and effective technique for producing green polymer composites [52]. Natural fibres are hand‐stacked on a mould coated with a release agent, specifically for mechanical properties. Thermoset resins, such as epoxy or polyester, are applied using brushes, rollers, or sprays to ensure complete fibre impregnation and to avoid retaining air bubbles, thus enhancing interfacial bonding and mechanical strength [53]. The physical properties of the final composites were further defined by appropriate curing at either ambient or elevated temperatures [54]. The hand‐lay‐on method can be employed on different bio‐based resins and fibres, making it suitable for large and complex structures in automotive, marine, and aerospace [55]. This process has unique advantages that make point‐of‐care testing one of the best processes for green composite manufacturing due to its ease of use, minimal waste, cost efficiency, and sustainable processing.

### 2.5 Resin Transfer Moulding

**FIGURE 4:**
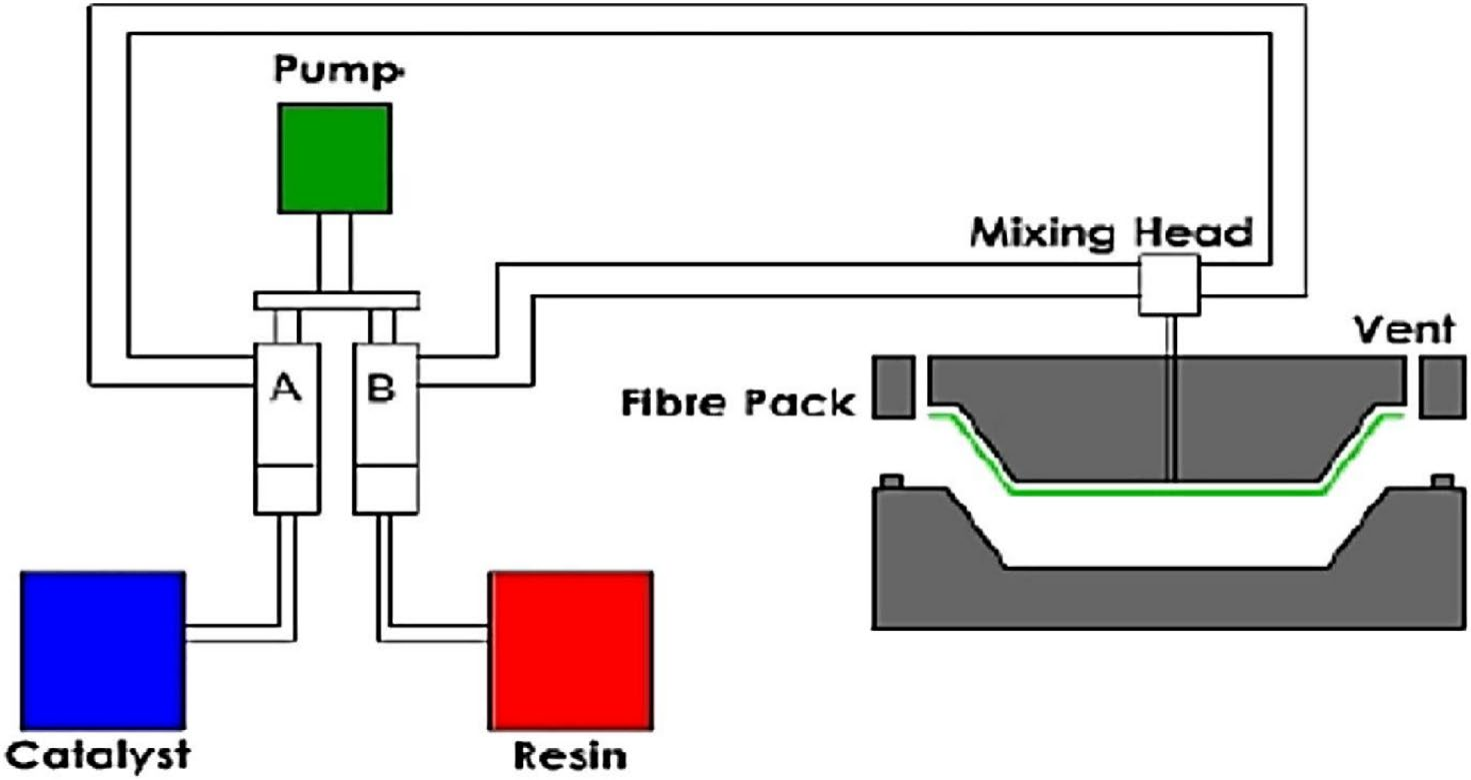
Schematic diagram of resin transfer moulding [57].

Zade et al. [56] described resin transfer moulding (RTM) as a closed‐mould process in which reinforcing fibres and synthetic and natural fibres are strategically positioned in the mould cavity before resin injection (see Figure 4). This fibre arrangement is critical because the orientation and distribution of fibres have a significant impact on the mechanical properties and overall performance of the resulting composite. Once the mould is sealed, heated liquid resin, typically a thermoset polymer, is injected under pressure to thoroughly saturate the fibres and fill any voids within the fibre architecture. By controlling the injection sequence, manufacturers can ensure thorough wetting of the fibres and minimize the risk of dry spots that could compromise the structural integrity of the composite [56].

Building on these basic principles, Mohammadabadi et al. [58] demonstrated the effectiveness of RTM in incorporating natural fibres into polymer matrices, resulting in environmentally friendly, high‐strength composites. Campos et al. [59] highlighted the importance of controlling the resin viscosity and moulding pressure to achieve uniform fibre impregnation. Precise control of these parameters reduces processing errors and ensures a robust fibre‐matrix bond, which is critical to the strength and durability of the composite. Once the fibres were fully saturated, the composite underwent a curing phase, which was performed under carefully monitored temperature and pressure conditions [60]. This curing step solidifies the resin, anchors the fibres in place, and gives the composite the final mechanical properties, including tensile strength, thermal stability, and impact resistance.

### 2.6 Additive Manufacturing Techniques

Additive manufacturing (AM), or 3‐dimensional (3D) printing provides a sustainable approach for producing customised polymer composites through layered material deposition. This enables precise fibre orientation and distribution that can enhance mechanical properties while also catering to user‐specific sectors, such as aerospace and automotive [61]. The strength and durability of fibre‐reinforced plastics are enhanced by improved interfacial bonding and fibre alignment, resulting from advancements in Fused Deposition Modelling (FDM). These advancements include in situ polymerisation and ex‐situ prepreg methods, which enable the potential integration of natural fibres [62].

## 3 Improving the Properties of Green Polymer Composites

In GPC, mechanical strength and thermal stability improve with the use of wooden, sisal, and agro‐waste natural fibres; however, fibre‐matrix adhesion often benefits only from the application of surface treatment. Nanoparticles such as boron nitride and copper oxide enhance thermal conductivity, barrier properties, and antimicrobial resistance [63]. PLA and Poly(butylene succinate) (PBS) form biodegradable polymer blends, but their mechanical properties remain competitive [64]. Meanwhile, hybrid reinforcement combining natural and synthetic fibres, such as glass or Kevlar, achieves improved tensile strength, impact resistance, and durability, making GPCs suitable for high‐performance sectors such as automotive and aerospace [65]. These strategies collectively facilitate the establishment of technically sound GPCs and support circular economic aspirations [66].

### 3.1 Fibre and Hybrid Reinforcement Approaches

Natural fibres are eco‐friendly alternatives to synthetic fibres used in GPC, offering enhancements in strength, stiffness, and durability [67]. These fibres provide a renewable and biodegradable source of reinforcement, effectively reducing the reliance on petroleum‐derived materials and lowering the carbon footprint of the composite. However, moisture absorption and poor compatibility with hydrophobic polymers hinder their reinforcement. Surface treatments such as alkali, silane, and plasma treatments improve fibre‐matrix adhesion [68]. A low fibre content results in poor reinforcement, whereas a high fibre loading may be poorly dispersed, decreasing the impact strength [69]. By selecting appropriate fibre types, implementing effective surface treatments, and optimising fibre content, GPCs can achieve mechanical properties comparable to and exceeding those of conventional synthetic composites.

Natural fibres can also be combined with synthetic materials or additives to create hybrid reinforcements that enhance the performance of composites. Embedding advanced integrated metal halide perovskite nanocrystals into polymer matrices improves their stretchability, stability, and processability [70]. The mechanical and thermal properties are significantly improved by hybrid woven structures that use biodegradable fibres such as PLA [18]. Agricultural waste, nanocellulose, and recycled fibres can be incorporated into GPCs, representing further optimisation. Silver nanoparticles embedded in sugar palm starch biopolymers enhance antibacterial functionality, whereas unbleached nanocellulose improves thermal stability [71]. Additionally, recycled industrial fibres offer a sustainable approach for achieving high performance in eco‐friendly applications [72]. Collectively, the fibre and hybrid reinforcement strategies improve GPC performance while supporting sustainability and the circular economy.

### 3.2 Nanoparticle Content in Green Polymer Composites

**FIGURE 5:**
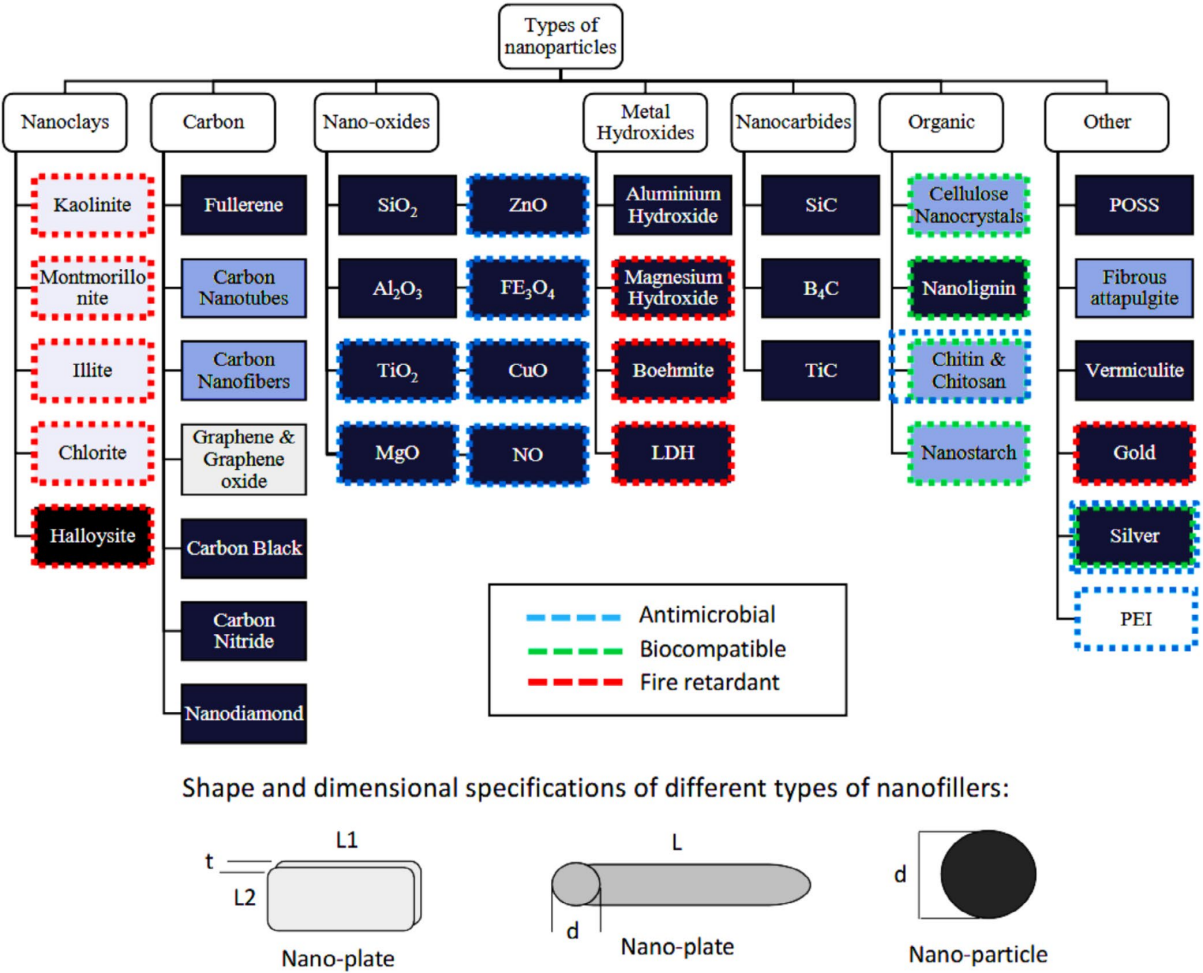
Types of nanofillers and the subcategories of each type. Dimensional and shape characteristics are highlighted in different shades of grey; the main application and properties are highlighted in coloured dashed lines [76].

The addition of nanoparticles to green polymer composites significantly enhances their mechanical, thermal, and functional properties while promoting environmental sustainability. Silver nanoparticles (AgNPs), synthesised through green methods utilising plant extracts exhibit enhanced antibacterial activity, optical properties, and material stability in polyvinyl alcohol (PVA) matrices [73]. The introduction of silica nanoparticles and surface‐modified silica significantly enhances the mechanical and thermal properties of biodegradable polymer composites, making them suitable for lightweight structural components and transparent coatings [74]. Advanced nanomaterials such as carbon nanotubes, graphene, and iron oxide (Fe_3_O_4_) play a significant role in electromagnetic shielding, thermal stability, and interfacial bonding. They effectively address void formation and enhance the overall performance of composites [75]. Nanoparticles used in green composites can be classified as nanoclays, carbon‐based materials, nano‐oxides, metal hydroxides, nanocarbons, and organics (Figure 5). Each type has its own functions; nanoclays and metal hydroxides are candidates for fire retardancy, carbon nanomaterials provide improved mechanical and electrical properties, and organic fillers such as chitin and cellulose contribute to biocompatibility. The symbols in the diagram represent the antimicrobial (blue), biocompatible (green), and fire‐retardant (red) characteristics for the targeted selection of specific applications.

### 3.3 Polymer Blend

Recent advances in green polymer composites have focused on blending various biodegradable polymers to improve material properties while maintaining sustainability. Polymers such as PLA, PHA, and starch‐based materials combined with natural fibres and nanomaterials are often valued for their degradability. Eryılmaz et al. [77] demonstrated the potential of natural fibres derived from *Allium fistulosum* L. (scallion), which have favourable tensile strength and thermal stability, making them ideal reinforcements for biodegradable polymer matrices. Blends with agricultural waste, as noted by Ncube et al. [78], have improved cost‐effectiveness and mechanical properties. Jo et al. [79] highlighted the inclusion of surface‐modified cellulose nanofibres (CNFs) in PHA matrices to improve interfacial adhesion and tensile strength. Yu et al. [80] explored hybrid mineral composites that combined PVA and sodium alginate (SA) with calcium phosphate oligomers, resulting in flexible and degradable materials suitable as replacements for traditional plastics. Khatri et al. [21] highlighted eco‐friendly electro‐spun hybrid nanofibres for energy and environmental use. These innovations support versatile and sustainable applications in various industries.

## 4 Applications of Green Polymer Composites

**FIGURE 6:**
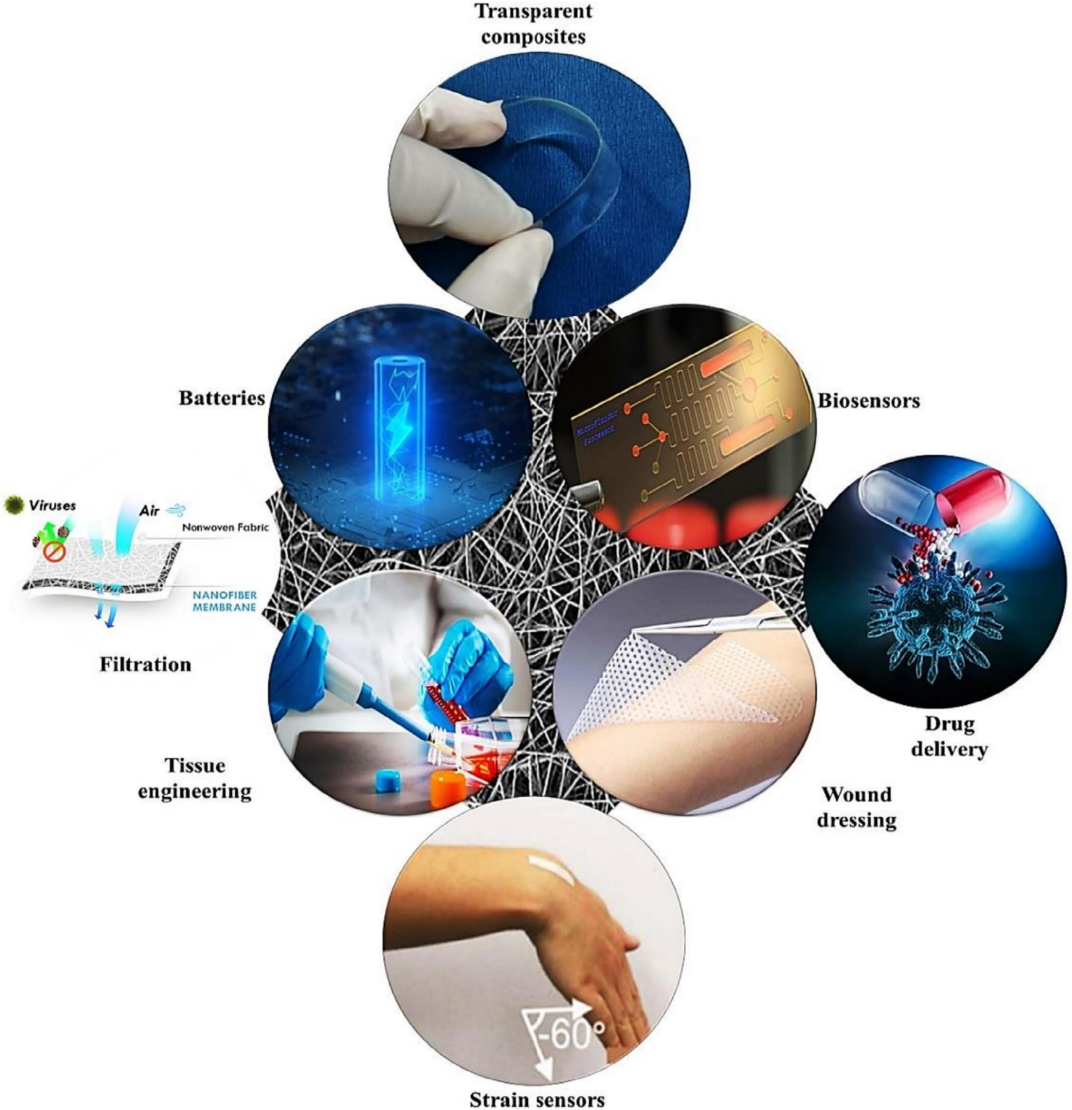
Notable areas for green polymer applications [81].

The increasing emphasis on sustainability has led to the widespread adoption of environmentally friendly polymer composites in various industries. These composites, which integrate natural fibres, biodegradable polymers, and innovative materials, offer environmentally friendly alternatives to traditional synthetic composites. Advances in materials science and processing techniques have made them versatile solutions for addressing structural, functional, and environmental challenges in critical sectors such as automotive, packaging, agriculture, medicine, and construction. Some notable areas of green polymer application are shown in Figure 6. Their application not only improves mechanical performance, but also reduces dependence on non‐renewable resources, thus minimizing waste and reducing CO_2_ emissions. Significantly, these applications also promote sustainability,   offer carbon footprint reduction, biodegradability, resource efficiency, and a circular economy. The widespread adoption of natural fibre composites in vehicles compromises vehicle structure, which reduces weight and fuel consumption, while biodegradable packaging materials shrink the impact of plastic pollution. By using these sustainable materials, industries can align themselves with global environmental goals while meeting the demand for high‐performance solutions.

**FIGURE 7:**
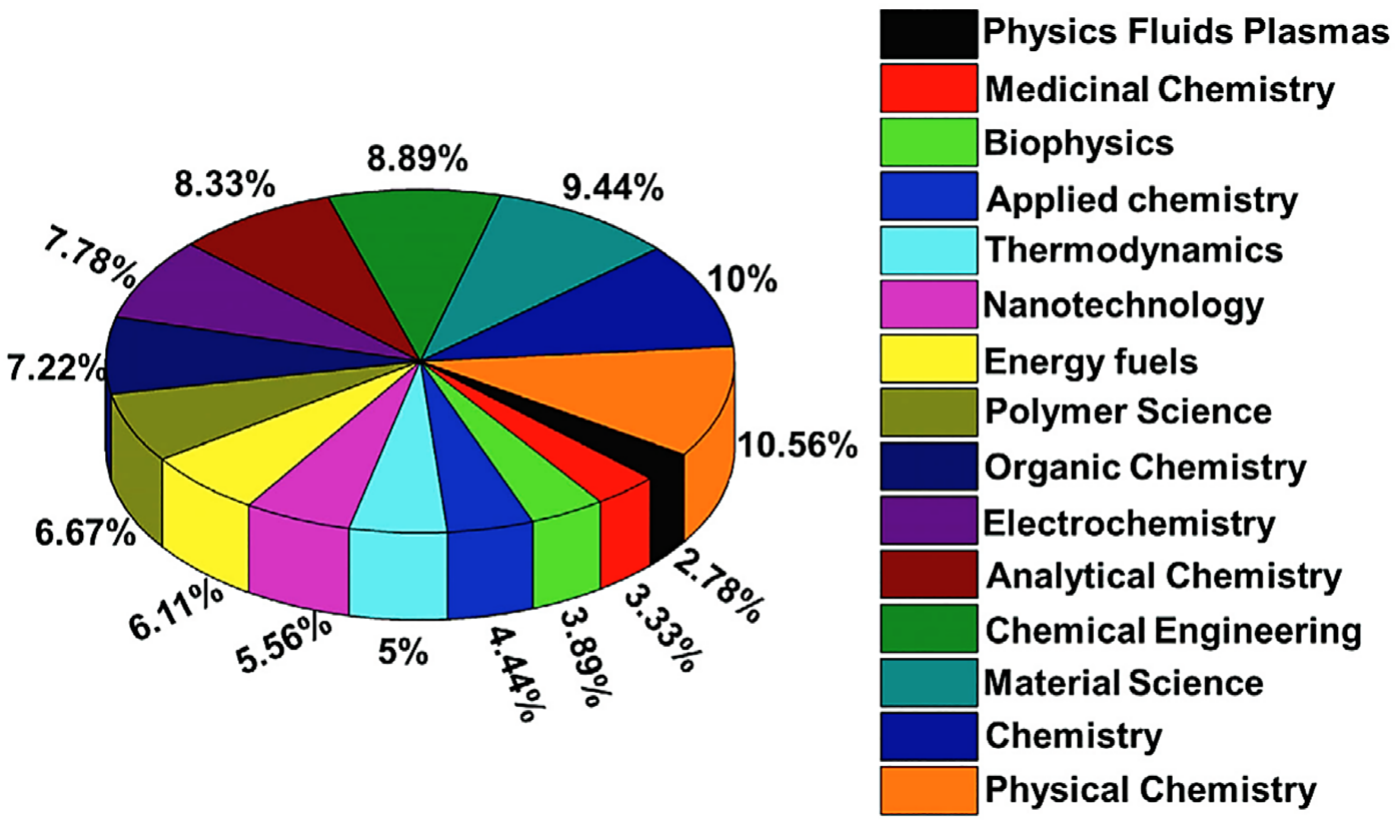
Classification of fields of interest and applications versus the number of publications [82].

The distribution of research efforts on green polymer composites across various applications has been well documented in the literature. Figure 7 illustrates this distribution and highlights the key focus areas. Mishra et al. [82] noted that many studies have emphasized biomedical applications, particularly in tissue engineering, drug delivery, and regenerative medicine. This emphasis is driven by the increasing demand for biodegradable and biocompatible materials in the health care sector. In addition, natural polymer‐based hydrogels, including chitosan and cellulose, have been examined for their antimicrobial properties, which make them suitable for wound dressings and scaffolds [83]. Environmental applications have also received considerable attention, particularly for biodegradable packaging and soil enhancement materials, reflecting heightened concerns regarding plastic pollution and sustainability. Studies have been dedicated to bioelectronics and energy production, in which green polymer composites provide environmentally friendly solutions for electronic devices [84]. These trends underscore the multidisciplinary nature of green polymer composites and address the critical global challenges related to healthcare, waste management, and environmental sustainability.

### 4.1 Automotive Industry

**FIGURE 8:**
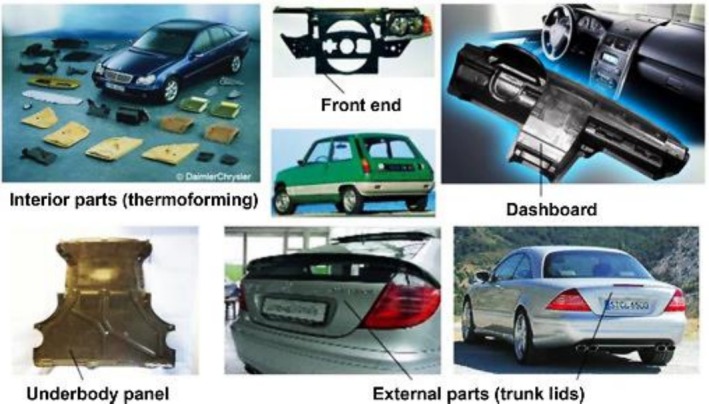
Applications of polymers in automobile parts [90].

Advancements in environment‐friendly green polymer composites have significantly influenced the automotive industry, particularly regarding vehicle weight reduction and fuel efficiency. Kalendova et al. [85] discussed the utilisation of clay‐based polymer nanocomposites for lightweight automotive components and highlighted their role in enhancing fuel efficiency and minimising environmental impact. These innovations highlight the necessity of preparing and characterising materials that fulfil the dual objectives of sustainability and performance in the automotive sector. Natural fibre composites (NFCs) have emerged as sustainable alternatives to synthetic materials in the automotive sector. Jariwala [86] emphasises the environmental advantages of NFCs, noting their lightweight nature, cost‐effectiveness, and versatility. Dzulkafly et al. [87] explored the application of empty oil palm fruit bunches in automobile interiors and demonstrated their commendable mechanical properties and environmental benefits. These materials illustrate how natural fibres can replace conventional plastics, reducing reliance on petrochemical products while aligning with sustainable infrastructure objectives. Alabtah et al. [88] highlighted the development of fibre‐reinforced systems designed to protect steel pipes from corrosion and emphasised durability as a critical factor in composite materials. These studies illustrate the potential of green polymer composites to replace conventional materials, balancing environmental and structural benefits in both sectors [89]. These advancements in the preparation and characterisation of high‐performance composites underscore their capacity to satisfy stringent environmental and engineering requirements in the automotive industry, thus justifying their application in various components, as shown in Figure 8.

### 4.2 Packaging Industry

**FIGURE 9:**
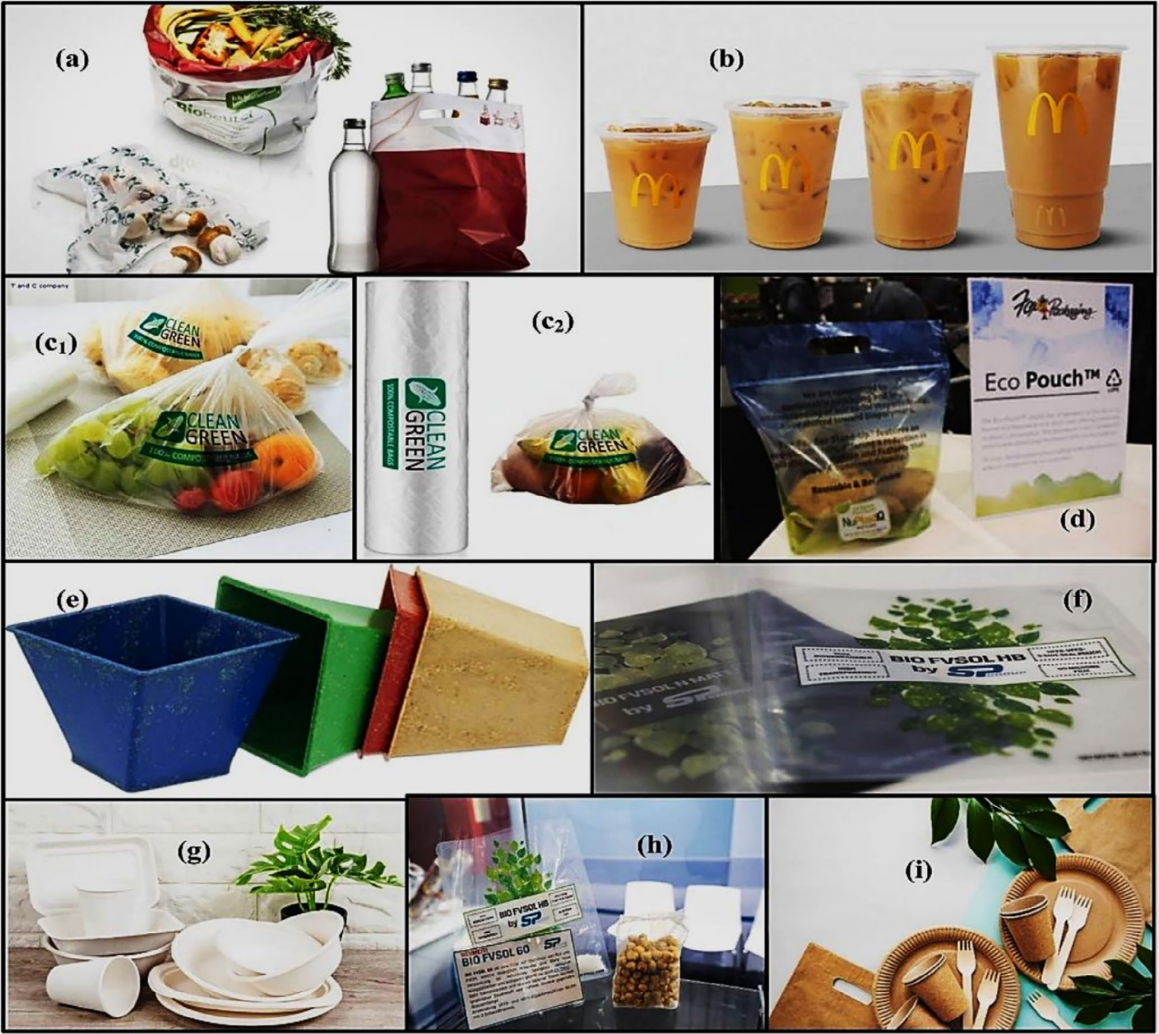
Green polymers for food packaging [94].

The transition to sustainable materials in the packaging industry is closely linked to the advances in environmentally friendly polymer composites. This change is driven by increasing consumer awareness and the demand for eco‐friendly solutions. Oliver et al. [91] highlight the growing consumer demand for sustainable food packaging and the urgent need for innovative materials that minimize environmental impact. This trend aligns with the increasing prioritization of sustainable materials in the construction sector. Similarly, Popovic et al. [92] investigated how consumers' knowledge of environmental issues and environmentally friendly lifestyles influences their willingness to adopt biodegradable packaging. Their findings highlight the importance of balancing product design and environmental awareness in order to facilitate market adoption. Numerous studies have highlighted the significant progress in the development of biodegradable polymer composites for packaging applications, emphasizing their potential to replace conventional plastics. Figure 9 shows various green polymer‐based materials used for food packaging. Belukhichev et al. [93] described environmentally friendly packaging films produced by blending polyvinyl chloride with biodegradable copolymers. This study illustrates how material composition can substantially reduce environmental impact.

Zeng et al. [95] investigated poly(vinyl alcohol)/kaolin barrier films and noted their superior mechanical properties and biodegradability. These innovations demonstrate how green polymer composites can enhance material performance while maintaining environmental benefits, thereby providing insights that can be applied to packaging. Natural and renewable resources have also emerged as focal points for the development of eco‐friendly packaging materials. Sari et al. [96] highlighted the use of bamboo in the Balinese souvenir industry, emphasizing its sustainability and alignment with eco‐design principles. Similarly, Tarique et al. [97] discussed arrowroot fibre‐reinforced biopolymer composites, which exhibited enhanced mechanical properties suitable for sustainable packaging applications. These examples illustrate the potential of natural fibres in creating biodegradable materials, highlighting their role in reducing plastic waste and promoting sustainable packaging practices. Altaf et al. [98] explored polysaccharide‐based bio‐nanocomposites, focusing on their environmentally friendly properties. Both studies emphasized the potential of these materials to replace conventional plastics in the packaging industry.

### 4.3 Agricultural Applications

Green polymer composites have emerged as promising alternative materials in the agricultural sector owing to their biodegradability and minimal environmental impact. Biodegradable polymers such as PLA, PHAs, PBS, and Poly(butylene adipate‐co‐terephthalate) (PBAT) are often used in mulch films, seed coatings, plant pots, and soil conditioners. These materials decompose over time and do not contribute to the plastic waste cycle or poor soil quality [99]. Green polymers have also promoted carbon‐neutral circular economic practices in agriculture [100]. By incorporating bioactive compounds and natural fillers into these materials, their mechanical and chemical properties can be significantly enhanced, making them suitable for demanding applications such as mulching and foliage protection [101]. PBAT composites exhibit a significant increase in strength, whereas CNF‐reinforced composites are utilized in packaging, as cellulose nanofibrils have gained popularity as a substitute for synthetic polymer additives [102]. Materials focused on innovative applications, such as controlled‐release fertilizers and superabsorbent hydrogels, are aimed at improving water retention, density, and nutrient mobility in drylands. Polymer‐photosensitizer composites also facilitate on‐demand biodegradability [103]. These contributions align with global sustainability goals and enhance agricultural efficiency.

### 4.4 Medical Applications

**FIGURE 10:**
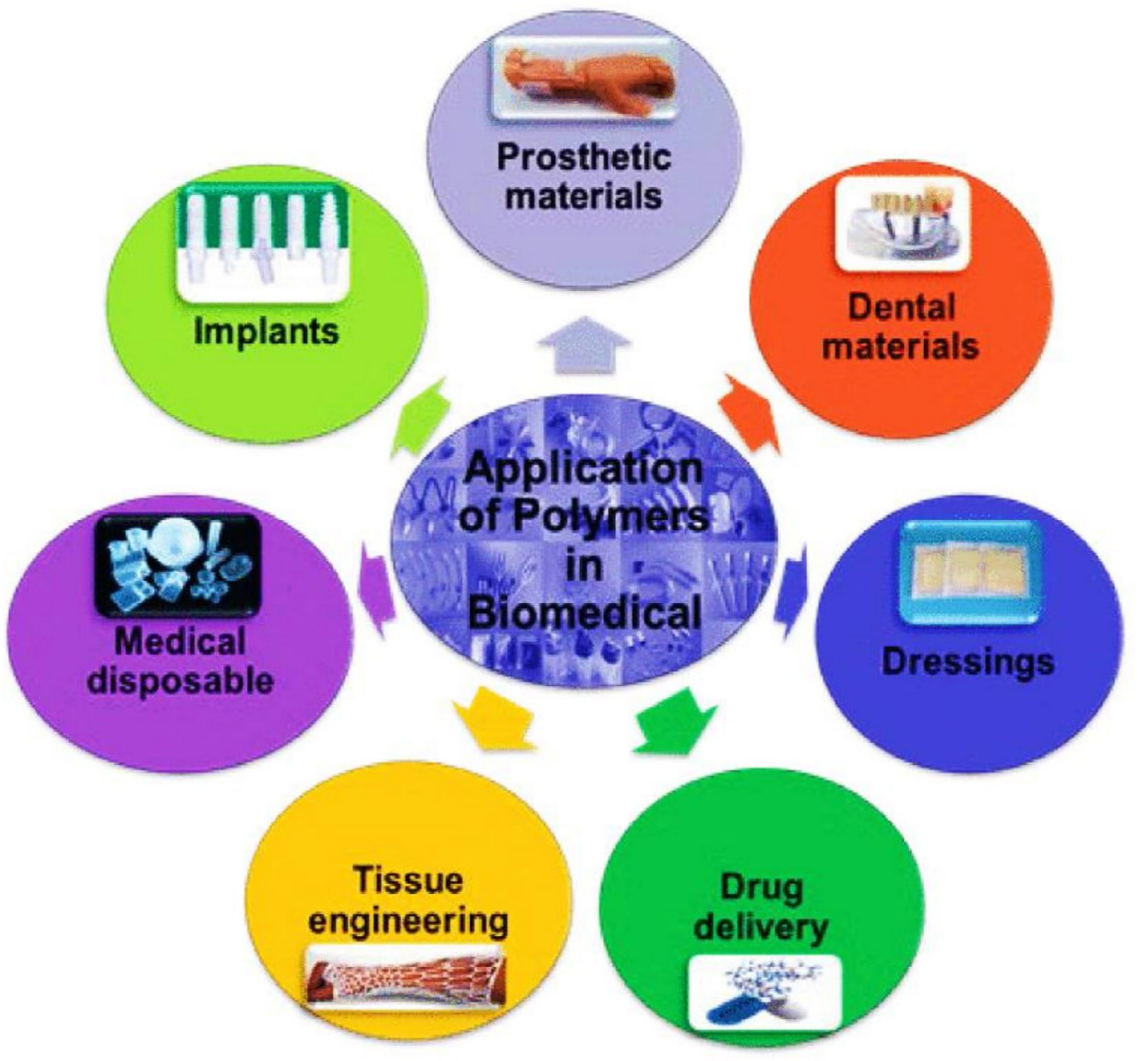
Green polymers used in biomedical applications [108].

Recent advances in green polymer composites have significantly transformed their medical application by providing sustainable, biocompatible, and high‐performance materials. As shown in Figure 10, these materials have been widely used in various medical applications. Kang et al. [104] explored polymer‐infiltrated ceramic network composites for dental restorations that effectively mimicked the structure and function of human tooth enamel and dentin. These composites not only represent a sustainable alternative to traditional materials but also have potential applications in healthcare infrastructure, including medical construction. Similarly, Koski et al. [105] demonstrated the effectiveness of starch‐hydroxyapatite composites in bone scaffolds and highlighted how natural polymers can improve biocompatibility and mechanical performance in bone and tissue engineering. Biodegradable and biocompatible hydrogels are also becoming increasingly important in medical applications, particularly in wound dressings and tissue scaffolds. As described by Pan et al. [106], chitosan and cellulose‐based hydrogels exhibit remarkable antimicrobial properties and environmental benefits in response to the increasing demand for sustainable solutions in the healthcare sector. In addition, black phosphorus and cellulose hydrogels have been investigated for cancer therapy and tissue scaffold construction, demonstrating their versatility in therapeutic and infrastructural applications [107]. Such materials combine bioactivity and degradability, thereby improving their suitability for biomedical applications.

The incorporation of biodegradable polymers further improves the functionality and environmental benefits of medical‐grade composites. Enzymatic polymerisation and selectively biodegradable polyesters have been investigated for applications in therapeutic devices, drug delivery systems, and tissue engineering [109]. Kenry et al. [84] investigated biodegradable conductive polymers for bioelectronics and regenerative medicine, highlighting their ability to combine functionality with ecological responsibility. Advanced techniques, such as the use of ionic covalent organic framework nanoparticles in PLA matrices, imparted antibacterial properties while maintaining thermal stability, making them suitable for biomedical devices [110].

### 4.5 Construction Industry

Natural fibres, such as jute, hemp, and sisal, are increasingly utilised as reinforcements in green polymer composites for construction owing to their mechanical strength, biodegradability, and positive environmental impact  [111]. The composite performance was further improved with agricultural wastes, such as rice husks and date palm fibres, which promoted both waste reduction and cost‐effectiveness [112]. These materials have contributed to the transition towards bio‐based, energy‐efficient building materials, offering advantages such as improved thermal insulation. The incorporation of renewable resources into fibre‐reinforced polymers enhances the load‐bearing capacity and durability of composite structures [113]. Green composites also contribute to sustainable architecture by employing environmentally friendly materials for both the structural and ornamental components [114]. This versatility makes green‐building qualifications suitable for various applications.

## 5 Life Cycle Assessment of Green Polymer

LCA is an essential methodology for evaluating the environmental impacts of 3D‐printed GPCs throughout their life cycle, including raw material extraction, manufacturing, usage, and end‐of‐life (EoL) disposal or recycling. Although GPCs are increasingly regarded as sustainable alternatives to traditional materials, this LCA study aims to accurately assess the environmental benefits and trade‐offs associated with their application as coatings. Recent research has highlighted the environmental advantages of natural fibre‐reinforced composites, particularly in EoL applications within the automotive sector, where the lightweight properties of these composites lead to fuel savings and reduced emissions. The incorporation of renewable feedstocks in bio‐composite matrices further minimises the carbon footprint and enhances sustainability [115]. However, economic considerations remain concerning the scalable production and cost‐effectiveness of post‐treatment processes. LCA studies underscore the potential of utilising local agricultural waste to enhance both environmental and social benefits. It is important to consider the disparities between energy consumption and greenhouse gas emissions arising from various moulding compositions and enhancement techniques [116]. EoL management is particularly significant; the environmental impacts of PLA–PP composites are significantly higher when sent to landfills than when recycled [117]. To fully realise the potential of LCA, future efforts should focus on ensuring high‐quality, standardised data and adopting a system thinking approach that effectively identifies critical environmental hotspots throughout a product's life cycle. Comprehensive LCA frameworks with robust EoL scenarios are essential for the development of policy‐driven material standards and facilitating large‐scale industrial integration of GPCs [118, 119]. In summary, while GPCs demonstrate considerable environmental performance, a thorough LCA is vital for a complete assessment of their sustainability potential and for informed implementation within the industry.

## 6 Conclusion and Future Trends

Green polymer composites (GPCs) are promising alternative sustainable materials that can outperform synthetic composites in various fields such as construction, automotive, medicine, and packaging. These composites can be created using advanced nanomaterials, natural fibres, and biodegradable polymers to improve their environmental performance, durability, and mechanical properties. However, several aspects of GPC projects pose challenges related to cost, scalability, and regulatory compliance that require further investigation. Future research should focus on hybrid reinforcements that combine natural and synthetic fibres, employ advanced nanotechnology to create structure‐oriented interfaces, and utilise AI‐driven processing and material formula optimisation. Establishing economic viability through optimised production processes and supply chains is essential, supported by comprehensive LCAs to validate environmental benefits. Moreover, strong standardisation frameworks, complementary policy incentives, and thorough sustainability certifications are crucial for overcoming commercialisation challenges and facilitating the adoption of GPCs across various sectors. With ongoing innovation, GPCs have the potential to contribute significantly to a circular economy and mitigate the environmental impacts across various industries. The study concludes, based on several reports analysed, that it is possible to promote the adoption of green polymer‐based composites without compromising the desired properties based on the intended application.

## Author Contributions

**Kobe Samuel Mojapelo:** conceptualisation, formal analysis, methodology, project administration, validation, writing the original draft, writing review and editing. **Williams Kehinde Kupolati:** formal analysis, methodology, validation, writing review, and editing. **Everardt Andre Burger:** methodology, validation, writing review and editing. **Julius Musyoka Ndambuki:** formal analysis, methodology, writing review and editing. **Jacques Snyman:** formal analysis, writing review and editing. **Emmanuel Rotimi Sadiku:** formal analysis, methodology, validation, writing review and editing.

## Ethics Statement

The authors have nothing to report.

## Conflicts of Interest

The authors declare no conflicts of interest.

## Data Availability

The data that support the findings of this study are available from the corresponding author upon reasonable request.
